# Antitumor effect of combined NAMPT and CD73 inhibition in an ovarian cancer model

**DOI:** 10.18632/oncotarget.6502

**Published:** 2015-12-08

**Authors:** Giovanna Sociali, Lizzia Raffaghello, Mirko Magnone, Federica Zamporlini, Laura Emionite, Laura Sturla, Giovanna Bianchi, Tiziana Vigliarolo, Aimable Nahimana, Alessio Nencioni, Nadia Raffaelli, Santina Bruzzone

**Affiliations:** ^1^ Department of Experimental Medicine, Section of Biochemistry, and CEBR, University of Genova, 16132 Genova, Italy; ^2^ Laboratorio di Oncologia Istituto G. Gaslini, 16147 Genova, Italy; ^3^ Department of Agricultural, Food and Environmental Sciences, Polytechnic University of Marche, 60131 Ancona, Italy; ^4^ Animal Facility, IRCCS AOU San Martino - IST Istituto Nazionale per la Ricerca sul Cancro, 16132 Genova, Italy; ^5^ Service and Central Laboratory of Hematology, University Hospital of Lausanne, 1011-CHUV, Lausanne, Switzerland; ^6^ Department of Internal Medicine, University of Genova, 16132 Genova, Italy; ^7^ IRCCS A.O.U. San Martino IST, Istituto Nazionale per la Ricerca sul Cancro, 16132 Genova, Italy

**Keywords:** NAMPT, CD73, NAD^+^, ovarian cancer

## Abstract

Nicotinamide phosphoribosyltransferase (NAMPT) is a crucial enzyme in the biosynthesis of intracellular NAD^+^. NAMPT inhibitors have potent anticancer activity in several preclinical models by depleting NAD^+^ and ATP levels. Recently, we demonstrated that CD73 enables the utilization of extracellular NAD^+^/nicotinamide mononucleotide (NMN) by converting them to Nicotinamide riboside (NR), which can cross the plasmamembrane and fuel intracellular NAD^+^ biosynthesis in human cells. These processes are herein confirmed to also occur in a human ovarian carcinoma cell line (OVCAR-3), by means of CD73 or NRK1 specific silencing. Next, we investigated the anti-tumor activity of the simultaneous inhibition of NAMPT (with FK866) and CD73 (with α, β-methylene adenosine 5′-diphosphate, APCP), in an *in vivo* human ovarian carcinoma model. Interestingly, the combined therapy was found to significantly decrease intratumor NAD^+^, NMN and ATP levels, compared with single treatments. In addition, the concentration of these nucleotides in ascitic exudates was more remarkably reduced in animals treated with both FK866 and APCP compared with single treatments. Importantly, tumors treated with FK866 in combination with APCP contained a statistically significant lower proportion of Ki67 positive proliferating cells and a higher percentage of necrotic area. Finally, a slight but significant increase in animal survival in response to the combined therapy, compared to the single agents, could be demonstrated. Our results indicate that the pharmacological inhibition of CD73 enzymatic activity could be considered as a means to potentiate the anti-cancer effects of NAMPT inhibitors.

## INTRODUCTION

Cells utilize nicotinamide adenine dinucleotide (NAD^+^) either as a coenzyme in redox reactions or as a substrate for NAD^+^-degrading enzymes, such as poly(ADP-ribose) polymerases (PARPs), ectocellular NAD^+^ases, including CD38 and sirtuins (SIRT1–7) [1]. Previously, our group reported that CD73, mostly known as the ectoenzyme dephosphorylating extracellular AMP to adenosine, is in fact also capable of degrading both NAD^+^ and NMN: specifically, CD73 degrades NAD^+^ to NMN and AMP, which are subsequently dephosphorylated by the same enzyme to NR and adenosine, respectively [2].

NAD^+^ metabolism plays a crucial role in the fate of tumor cells and cellular NAD^+^ levels are the result of a fine balance between opposite processes of NAD^+^ synthesis and degradation.

In mammalian cells, NAD^+^ is mainly synthesized from nicotinamide (NAM) through a salvage pathway sequentially involving the enzymes NAM phosphoribosyltransferase (NAMPT), which produces NMN, and NMN adenylyltransferase, which synthesizes NAD^+^ from NMN and ATP [3–5]. NAMPT is overexpressed in different types of cancer, including prostate, gastric, breast and ovarian cancer, gliomas, leukemia, lymphoma and myeloma [6, 7]. The mechanism by which NAMPT is fundamental in tumor progression certainly relies on the fact that NAD^+^ is required for the activity of NAD^+^-metabolizing enzymes, such as PARP1, SIRT1, SIRT6 and CD38, all having different cancer-promoting abilities, such as genomic stability, secretion of pro-inflammatory cytokines, angiogenesis, motility and invasion [8–11].

NAMPT inhibitors, including FK866 and CHS828, have potent anticancer activity in several preclinical models of solid and hematologic cancers [6] by depleting NAD^+^ and ATP levels and thereby leading to tumor growth inhibition [6–8, 12–15].

Despite their promise in preclinical models, in phase I/II clinical studies, FK866 and CHS828 essentially failed to show signs of a meaningful anticancer activity in patients with solid tumors [16]. Regarding safety of these compounds, the main forms of reported toxicity were thrombocytopenia, various gastrointestinal symptoms and lymphopenia [6]. Also based on these results, currently, there is a general agreement on the fact that NAMPT inhibitors should be administered in combination with other treatments, to increase their efficacy [6, 8]. One suggested possibility is the combination of NAMPT inhibitors with PARP-activating chemotherapeutics, which should further enhance the decrease in intracellular NAD^+^ content [17, 18]. Based on an opposite rationale, NAMPT inhibition has also been proposed to synergize with PARP inhibitors in triple-negative breast cancer [19]. NAMPT inhibition was also found to increase the efficacy of Tumor Necrosis Factor-Related Apoptosis Inducing Ligand (TRAIL), HDAC inhibitors, P-glycoprotein-1 (Pgp) inhibitors and of Rituximab, an anti-CD20 antibody, in leukemia cells [20–23]. Finally, NAMPT inhibition enhanced the effect of radiation in an *in vivo* prostate cancer model [24].

A plausible explanation for the limited activity of single-agent NAMPT inhibitors as cancer therapeutics in clinical trials could be the presence in human body fluids of NAD^+^ or NAD^+^ precursors, including nicotinic acid (NA), NMN and NR, which could well substitute for the inhibited NAD^+^ biosynthesis from NAM [25]. Indeed, NAD^+^ and NMN have been detected in mammal plasma and fluids [26, 27]. NAD^+^ efflux from cells can occur through a non-specific cell death, or through Cx43 hemichannels, with a regulated mechanism [28, 29].

Recently, we demonstrated that endogenous CD73 enables the utilization of extracellular NAD^+^/NMN as a precursor for intracellular NAD^+^ biosynthesis in human cells by converting NAD^+^/NMN to NR which, in turn, can cross the plasma membrane and be phosphorylated intracellularly to NMN [25]. In *in vitro* cell systems, we demonstrated that when CD73 is either silenced or pharmacologically inhibited, the salvage of FK866-treated cells by extracellular NMN is reduced [25].

An increased CD73 expression has been observed in several types of cancer, and the tumor microenvironment contains factors promoting CD73 expression [30]. High CD73 expression and activity confer a survival advantage to cancer cells, frequently determining metastasis and a poor prognosis [31]. Up to now, however, CD73 role in cancer has always been ascribed to the CD73-mediated extracellular production of adenosine and to the regulation of purinergic receptor activity [32–35]. Indeed, CD73 is considered an appealing therapeutic target for treating cancer and the CD73 inhibitor α, β-methylene adenosine 5′-diphosphate (APCP) shows promising anticancer activity, by inhibiting CD73-mediated functions in tumor cells and in T-cell immunity [33, 36, 37].

Epithelial ovarian cancer (EOC) is the most lethal gynecologic malignancy worldwide, with a 5-year survival of less than 30% for the women diagnosed at advanced stage [38]. Thus, advances in the identification of new therapeutical strategies are demanded.

**Figure 1:**
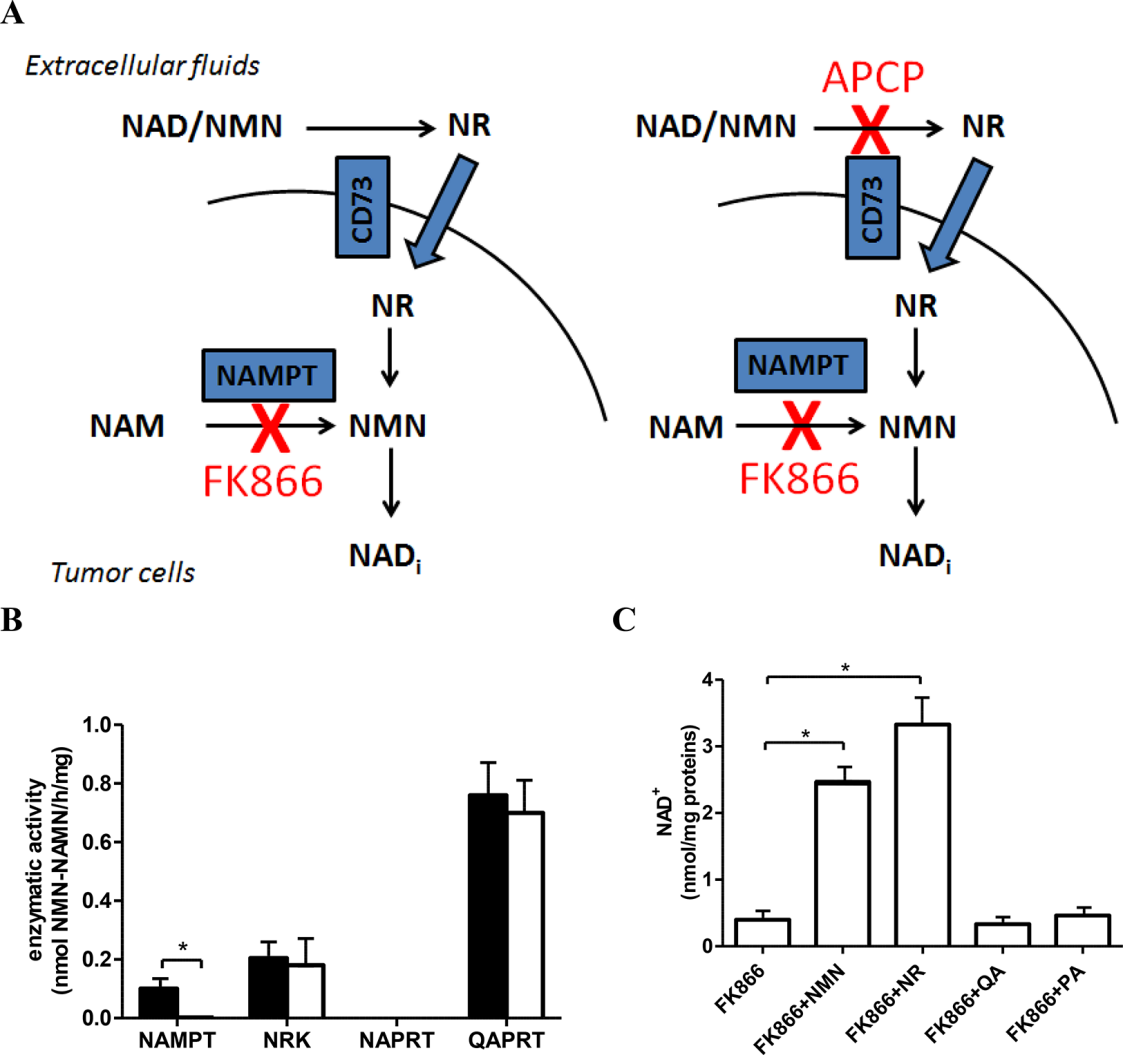
OVCAR-3 cells are sensitive to NAMPT inhibition with FK866 (**A**) Schematic representation of the rationale for simultaneously inhibiting CD73 and NAMPT. (**B**) Screening of the NAD^+^ biosynthetic enzyme activities performed on cell lysates from untreated OVCAR-3 cells (black bars) or from cells treated for 24 h with 30 nM FK866 (white bars). Results are mean ± SD of 3 determinations. *p < 0.001. (**C**) OVCAR-3 cells were incubated for 48 h with 30 nM FK866, in the presence of 10 μM NMN, NR, QA or of 100 μM PA. Results are mean ± SD of at least 3 determinations. *p < 0.01 compared to FK866 alone.

Here, we investigated the anti-tumor potential of simultaneously inhibiting NAMPT (with FK866) and CD73 (with APCP) in an *in vitro* and *in vivo* human ovarian carcinoma model. Blocking CD73 would hamper the generation of NR to be used as an intracellular NAD^+^ precursor by cancer cells, thereby leading to a marked potentiation of FK866 anticancer effects (Figure 1A).

## RESULTS

### Identification of the NAD^+^ biosynthetic pathways in OVCAR-3 cells

The ovarian cancer cell line OVCAR-3 is an established model for *in vivo* studies of cancer therapeutics: their intraperitoneal inoculation leads to a local dissemination with formation of tumor masses and ascites, in which it is possible to measure the amount of extracellular metabolites. To verify that OVCAR-3 cells represented an appropriate model for our study, we preemptively assessed i) the NAD^+^ biosynthetic pathways that are active in these cells, ii) their sensitivity to FK866, and iii) the expression of CD73.

Since NAD^+^ can be synthesized from various precursors and through different pathways [5], we screened which of these pathways are present in OVCAR-3 cells. Specifically, the activities of NAMPT (converting NAM to NMN), nicotinamide riboside kinase (NRK; phosphorylating NR to NMN), and of nicotinate phosphoribosyltransferase (NAPRT; converting NA to NAMN), were measured in OVCAR-3 cell extracts. In addition, the activity of quinolinate phosphoribosyltransferase (QAPRT), involved in the de-novo synthesis of NAD^+^ from tryptophan, was also tested. As shown in Figure 1B, both NAMPT and NRK activities could be detected in OVCAR-3 cells, indicating that these cells are able to use both NAM and NR as NAD^+^ precursors. In humans, NRK activity is expressed by two different isoforms, which can be enzymatically distinguished based on kinetic parameters [39]. The fact that the NRK activity in OVCAR-3 cell lysates, as assessed in the presence of 72 μM NR, was similar to the one measured in the presence of 36 or 18 μM NR, i.e. below the K_m_ for NR of the NRK2 isoform (46 μM, ref. 39), indicates that the main NRK activity is due to the expression of NRK1 (K_m_ for NR, 3.4 μM, ref. 39) (Supplementary Figure S1). When OVCAR-3 cells were incubated for 24 h in the presence of FK866, NAMPT activity was almost completely inhibited, whereas the other enzymatic activities were not affected (Figure 1B): thus, NAMPT inhibition with FK866 does not alter the enzymatic activity of the other enzymes involved in NAD^+^ synthesis. The intracellular NAD^+^ concentration was greatly reduced by a 48 h-incubation with 30 nM FK866 (from 19.12 ± 4.88 to 0.40 ± 0.12 nmol/mg proteins), indicating a major role for the NAMPT-mediated NAD^+^ production in OVCAR-3 cells. The exogenous addition of NMN or NR reverted FK866-induced NAD^+^ reduction (Figure 1C). NAPRT activity was not detectable in these cell extracts, whereas QAPRT was the most abundant activity (Figure 1B). The de-novo route controlled by QAPRT is considered not relevant in most cell types, under physiological conditions [5]. To analyze the contribution of QAPRT activity to NAD^+^ maintenance in OVCAR-3 cells, cells were incubated in the presence of quinolinic acid (QA). No increase in their intracellular NAD^+^ concentration was observed in control (20.80 ± 3.52 nmol/mg proteins) or in FK866-treated cells (Figure 1C). Accordingly, addition of phthalic acid (PA), a QAPRT inhibitor, failed to affect the intracellular NAD^+^ levels under either type of treatment conditions (control+ phthalic acid: 20.98 ± 2.87 nmol/mg proteins; FK866+ phthalic acid: Figure 1C). All together, these results indicate that in OVCAR-3 cells QAPRT does not significantly contribute in maintaining NAD^+^ levels. The significance of the marked QAPRT activity measured in the lysate of these cells remains to be elucidated. The possibility that during the preparation of the lysate an inhibitory factor of the enzyme has been removed or diluted cannot be ruled out, as occurred in [40]. In this view, in ovarian carcinoma cells, QAPRT activity might become relevant to NAD^+^ production only under specific, still unknown conditions. For instance, it has been reported that neoplastic transformation in astrocytes is associated with a QAPRT-mediated switch in NAD^+^ synthesis, whereby glioma cells begin to utilize microglia-derived QA as an alternative NAD^+^ precursor thereby acquiring resistance to radiochemotherapy [41].

### CD73 inhibition reduces the NMN-mediated rescue of FK866-induced death in OVCAR-3 cells

**Figure 2:**
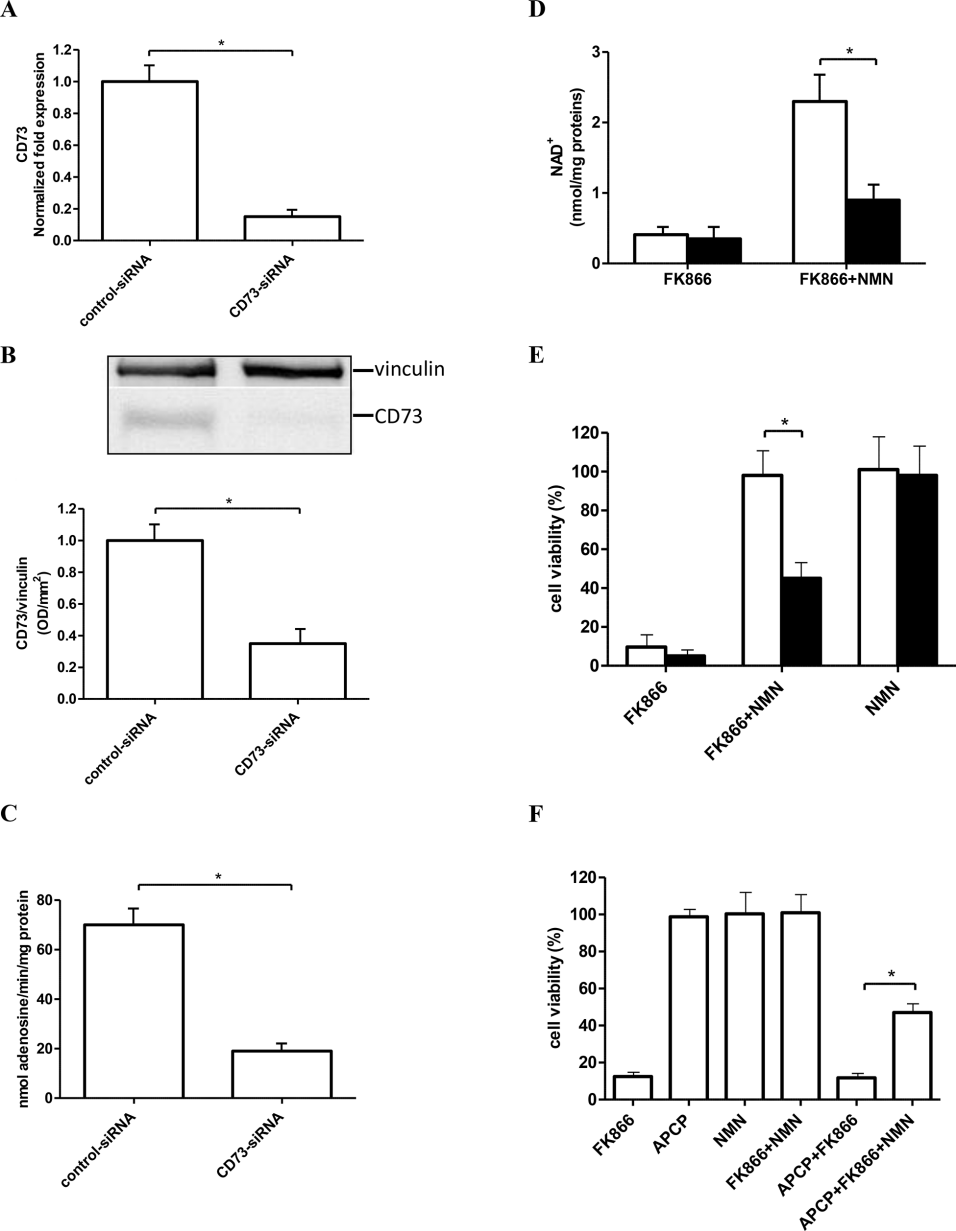
Silencing of CD73 expression or pharmacological CD73 inhibition impairs the NMN-mediated rescue from FK866-induced cell death (**A–E**) OVCAR-3 cells were transfected in the presence of a specific CD73-siRNA or negative control-siRNA (control-siRNA). Twenty four hours after transfection: qPCR analysis (A), Western blot analysis (B), and evaluation of the AMP-degrading activity (C) were performed; (D–E) After 24 h after electroporation, cells transfected with control-siRNA (white bars), or with CD73-siRNA (black bars), were treated (or not) with 30 nM FK866, in the presence or absence of NMN (added twice/day at 10 μM final concentration) and NAD^+^ content (D) and cell viability (E) were evaluated after 48 and 72 h, respectively. Cell viability is expressed as % of untreated cells. Data are mean ± SD (*n* = 3). In panel B, one representative blot and the mean ± SD of 3 different analysis are shown *p < 0.05. (**F**). OVCAR-3 cell viability (5 × 10^3^ well in 96-well plates) was evaluated after 72 h incubation of the cells in complete medium with or without 30 nM FK866, in the presence or absence of 1 μM adenosine 5′-(α, β-methylene)diphosphate (APCP), or of 10 μM NMN (added twice a day). Results are expressed as percentage of cell growth relative to untreated, control cells. Data are expressed as mean ± SD (*n* = 3). *p < 0.05, compared to FK866^+^ NMN-treated cells.

Next, we assessed CD73 expression in OVCAR-3 cells. At first, we evaluated the presence of ectocellular AMP-degrading activity, which was 71.9 ± 12.1 nmol adenosine/min/mg protein, as measured using intact cells. A qPCR analysis also confirmed presence of CD73 mRNA in OVCAR-3 cells, which was reduced by 85% at 24 h after transfection with CD73-siRNA (Figure 2A). In accordance, Western blot analysis on cell lysates from OVCAR-3 cells, also revealed a band approximately at a 70 kDa molecular weight, which was decreased by 65% in cells transfected with CD73-siRNA (Figure 2B). CD73-silencing greatly reduced the AMP-degrading activity (Figure 2C), confirming that this enzymatic activity reflected the presence of CD73, and not of other enzymes. Finally, CD73 expression on intact cells was also demonstrated by FACS analysis (Supplementary Figure S2). Since extracellular NAD^+^/NMN might also be a substrate for CD38, being hydrolyzed to NAM [25, 42], we evaluated CD38 enzymatic activities in OVCAR-3 cells: however, no detectable production of NAM and ADPR from NAD^+^, nor of NAM and cyclic-GDP-ribose from NGD^+^ (a NAD^+^ analogue used to measure the CD38-mediated ADP-ribosyl cyclase activity, ref. 43) could be detected (data not shown).

We previously showed that, in U87 (human glioblastoma) and A549 (human lung) cells, CD73 silencing or its pharmacological inhibition with APCP prevented the rescue from FK866 cytotoxicity by NMN supplementation [25]. In line with this observation, CD73-silencing reduced by 70% the ability of FK866-treated OVCAR-3 cells to generate intracellular NAD^+^ using extracellular NMN (Figure 2D).

The presence of 30 nM FK866 for 72 h in the culture media greatly reduced (by approximately 90%) OVCAR-3 cell viability, and extracellular NMN completely rescued cells from the FK866-induced cell death (Figure 2E, control cells), as previously shown for other cell lines [25]. When CD73 expression was down-regulated, the NMN-mediated rescue of the FK866-induced cell death was significantly reduced (Figure 2E). Similar measurements of cell viability in the presence of the specific CD73 inhibitor APCP, indicated that APCP *per se* did not affect viability of control or of FK866-treated cells, whereas it significantly alleviated the protective effects of exogenously added NMN on FK866-treated malignant cells (Figure 2F). These data indicate that CD73 activity is necessary for utilization of extracellular NMN to fuel intracellular NAD^+^ synthesis and thereby rescue cells from the FK866-induced cell death.

**Figure 3:**
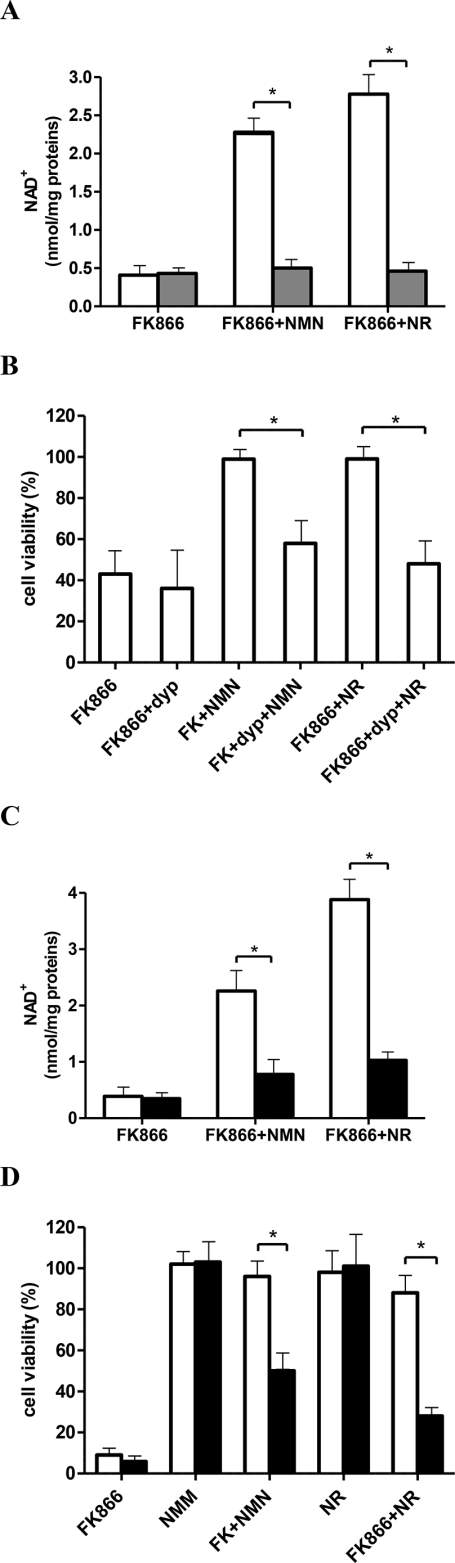
Inhibition of NR transport or silencing of NRK1 expression impairs the NMN-mediated rescue from FK866-induced NAD^+^ depletion and cell death (**A–B**) OVCAR-3 cells were incubated for 48 h in the presence of 30 nM FK866, with (grey bars) or without (white bars) 5 μM dipyridamole (dyp) and NMN or NR (added twice/day at 10 μM final concentration), as indicated. NAD^+^ content (A) and cell viability (B) were evaluated after 48 h. Cell viability was expressed as relative to control, untreated cells. (**C**, **D**) OVCAR-3 cells were transfected in the presence of a specific NRK1-siRNA (black bars) or negative control-siRNA (white bars). Twenty four hours after transfection, cells were treated (or not) with 30 nM FK866, in the presence or absence of NMN or NR (added twice/day at 10 μM final concentration): NAD^+^ content (C) and cell viability (D) were evaluated after 48 h and 72 h, respectively. Results are the mean ± SD of at least 3 independent determinations. *p < 0.05.

To conclusively demonstrate that NMN, in order to be utilized by cells, is converted into NR, which can cross the plasmamembrane through a dypiridamole-inhibitable nucleoside transporter [44], OVCAR-3 cells were incubated, or not, with FK866, NR, or NMN, with or without dypiridamole. Dypiridamole almost completely abrogated the NR-mediated rescue of FK866-treated cells and greatly inhibited the rescue obtained with NMN, both in terms of intracellular NAD^+^ content (Figure 3A) and of cell viability (Figure 3B). The effects of dypiridamole are in line with the idea that NR influx into cells has to occur to ensure cell rescue from FK866 by NMN (or NR itself). Finally, NRK1 expression was specifically silenced in OVCAR-3 cells: qPCR analysis performed 24 h after transfection with NRK1-specific siRNA demonstrated a 65 ± 15% (*n* = 3) reduction in NRK1 mRNA levels. In NRK1-silenced cells, both NR- and NMN-mediated rescue from FK866-induced NAD^+^ depletion (Figure 3C) and cell death (Figure 3D) were potently and significantly reduced.

### Combined treatment with NAMPT and CD73 inhibitors affects tumor NAD^+^, NMN and ATP content

The data obtained *in vitro* in OVCAR-3 cells suggested that this cell line was suitable for investigating whether the simultaneous administration of FK866 and APCP would result in a more robust reduction in the intracellular NAD^+^ content in an *in vivo* cancer model. Thus, an *in vivo* experiment was conducted utilizing athymic nude mice that were inoculated intraperitoneally with OVCAR-3 cells stably transfected with firefly-luciferase. The expression of firefly-luciferase by the cancer cells allowed us to monitor tumor development by *in vivo* bioluminescence using the IVIS™ Imaging System. Mice were randomized to the following treatment groups: vehicle, FK866, APCP or the combination of the two molecules. Animals were treated for 28 days: body weight measurements and general physical status recordings indicated the absence of major toxicities as a result of these treatments throughout the treatment period.

**Figure 4:**
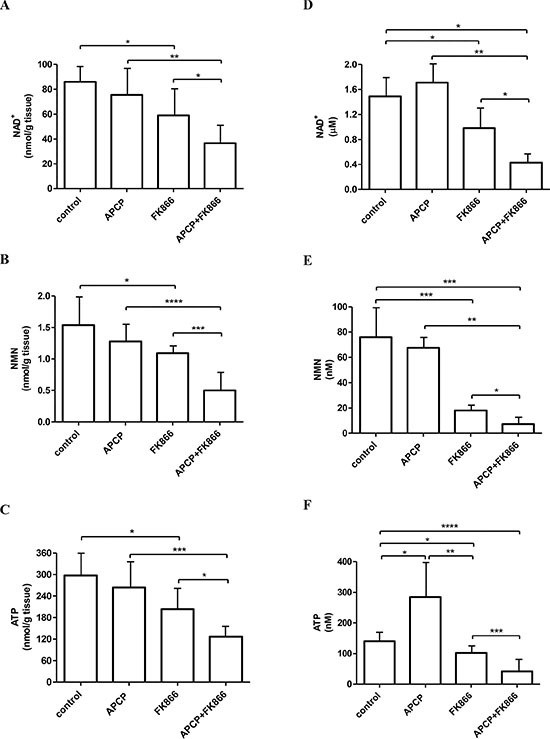
NAD^+^, NMN and ATP levels in tumors and in ascitic exudates from control and treated mice NAD^+^ (**A**, **D**), NMN (**B**, **E**) and ATP (**C**, **F**) levels were measured in tumors (A, B, C) or in ascitic exudates (D, E, F) recovered from the animals treated with vehicle (control), or FK866 or APCP or their combination. Results represent the mean ± SD (tumors, *n =* 8; ascitic exudates, at least *n =* 5). *p < 0.05; **p < 0.01; ***p < 0.001; ****p < 0.0001.

NAD^+^, NMN and ATP levels were evaluated both in the recovered tumor mass and in ascitic exudates. NAD^+^ content in the tumors was more pronouncedly decreased in animals treated with the combination of the two molecules than with FK866 alone (Figure 4A), confirming that NAMPT and CD73 inhibition affect two different pathways of intracellular NAD^+^ biosynthesis. Consistent with these data, NMN levels within tumor masses were found to be significantly reduced in the combination treatment arm, as compared to those measured in mice that had been treated with FK866 alone (Figure 4B), which is in keeping with the involvement of NMN as an intermediate metabolite of both NAMPT- and NRK-mediated pathways of NAD^+^ production (Figure 1A). Finally, intratumor ATP levels were also strongly decreased by the simultaneous inhibition of NAMPT and CD73 (Figure 4C), which is consistent with previous studies reporting that inhibition of NAD^+^ biosynthesis also affects ATP content [14, 45]. APCP alone did not significantly decrease intratumor NAD^+^, NMN and ATP levels, as compared to control animals (Figure 4), suggesting that, under baseline conditions, within a tumor, NAD^+^ is primarily synthesized from NAM.

Next, since the presence of extracellular NAD^+^ and/or NMN in the fluids surrounding the tumor represents a fundamental pre-requisite to verify the hypothesis that interfering with the utilization of extracellular NAD^+^/NMN by inhibiting CD73 activity could enhance the anti-tumor activity of FK866, we measured NAD^+^ and NMN levels in the ascitic exudates collected from the animals at sacrifice. Indeed, both NAD^+^ and NMN could be detected in the untreated controls (Figure 4D, 4E). In animals treated with the combination therapy, both NAD^+^ and NMN levels were strongly reduced, indicating that the levels of extracellular nucleotides indeed mirror the intracellular values (Figure 4D, 4E). In FK866-treated animals, NAD^+^ and, more remarkably, NMN levels were decreased compared to those measured in exudates from control animals (Figure 4D, 4E). ATP levels were significantly lower in exudates from animals treated with the combination therapy, as it was observed for the intracellular ATP content. The observation of higher ATP levels in APCP-treated animals (Figure 4F) could be explained by the absence of downstream AMP degradation to adenosine due to CD73 inhibition by APCP.

Interestingly, NR was indeed detectable in ascitic exudates, which is in agreement with the presence of the ectocellular CD73 activity in tumor cells. Low NR values, ranging from 3 nM to 68 nM, were measured in the tested samples, which might suggest a marked uptake of extracellular NR [44]. A high degree of variability in NR levels was observed within ascitic exudates within group of treatment, which might reflect NR values that were very close to the detection limit of our method. As a result, no comparison between the different treatments under this respect (effects on extracellular NR levels) was possible.

### Simultaneous administration of NAMPT and CD73 inhibitors differently affects NAD^+^ content in hearts and livers

**Figure 5:**
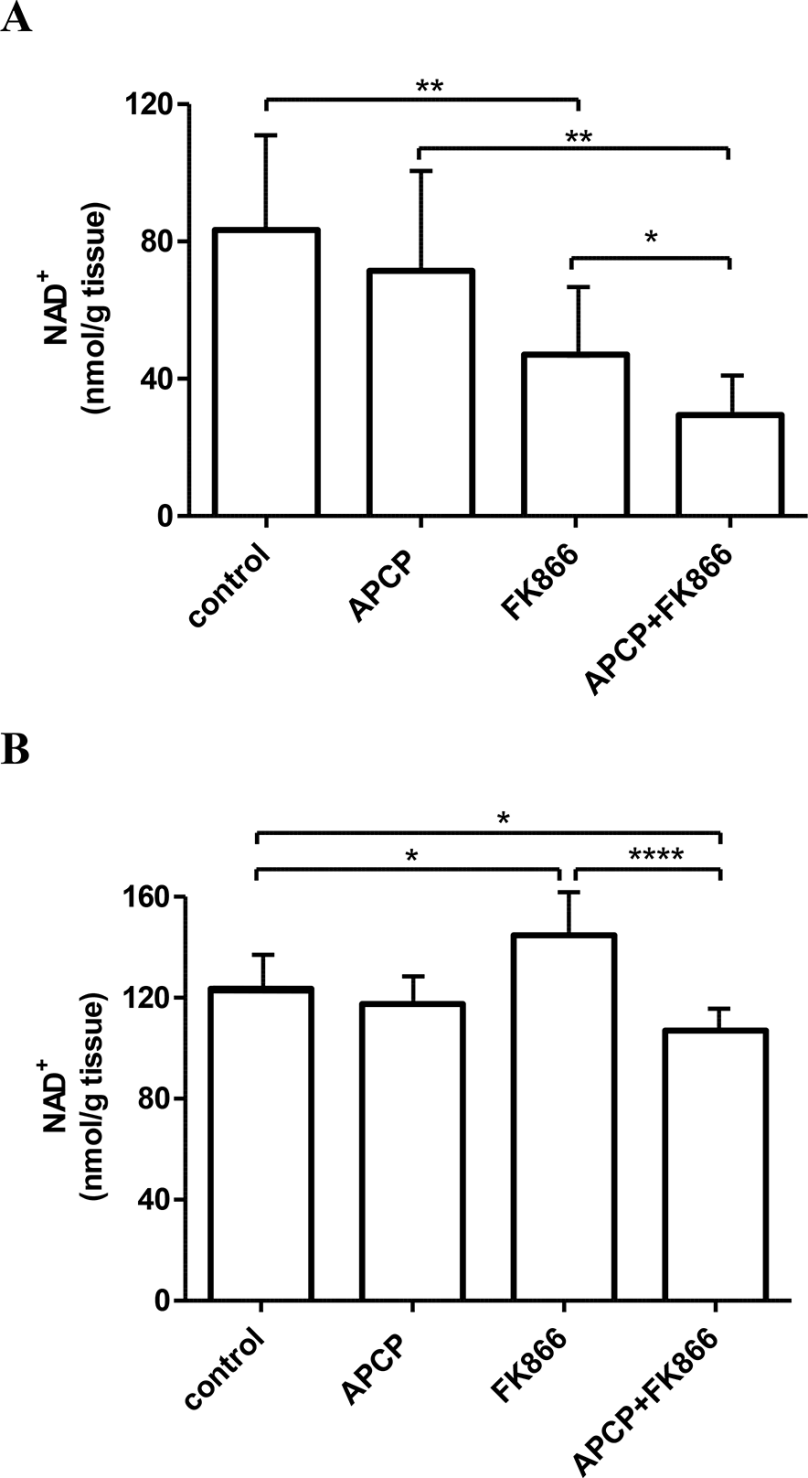
NAD^+^ levels in hearts and livers from control and treated mice NAD^+^ levels were measured in hearts (**A**) and livers (**B**) recovered from the animals treated with vehicle (control), or FK866 or APCP or their combination. Results are expressed as nmol/g tissue and represent the mean ± SD (*n =* 8). *p < 0.05; **p < 0.01; ****p < 0.0001.

The effects of the different treatments were also evaluated by measuring NAD^+^ levels in hearts and livers. As shown in Figure 5A, the pattern of NAD^+^ levels in the heart after the different treatments essentially paralleled the NAD^+^ levels that were detected in the tumors. FK866 treatment significantly reduced intracellular NAD^+^ levels in hearts (Figure 5A), in line with a previous report [46]. Interestingly, CD73 activity appeared to be relevant for the maintenance of intracellular NAD^+^ content in the heart, too, since the simultaneous inhibition of NAMPT and CD73 decreased heart NAD^+^ levels to a higher extent as compared to NAMPT inhibition alone (Figure 5A).

In contrast with what observed in tumors and hearts, treatment with FK866 increased NAD^+^ levels in the liver (Figure 5B). This observation is in agreement with results obtained with a different NAMPT inhibitor (administered to mice for 5 days), which also did not reduce liver NAD^+^ content [47]. However, simultaneous inhibition of CD73 and NAMPT did significantly reduce (by approximately 25%) liver NAD^+^ content as compared to the NAD^+^ content measured in FK866-treated animals.

### *In vivo* antitumor effect of NAMPT and CD73 inhibition

**Figure 6:**
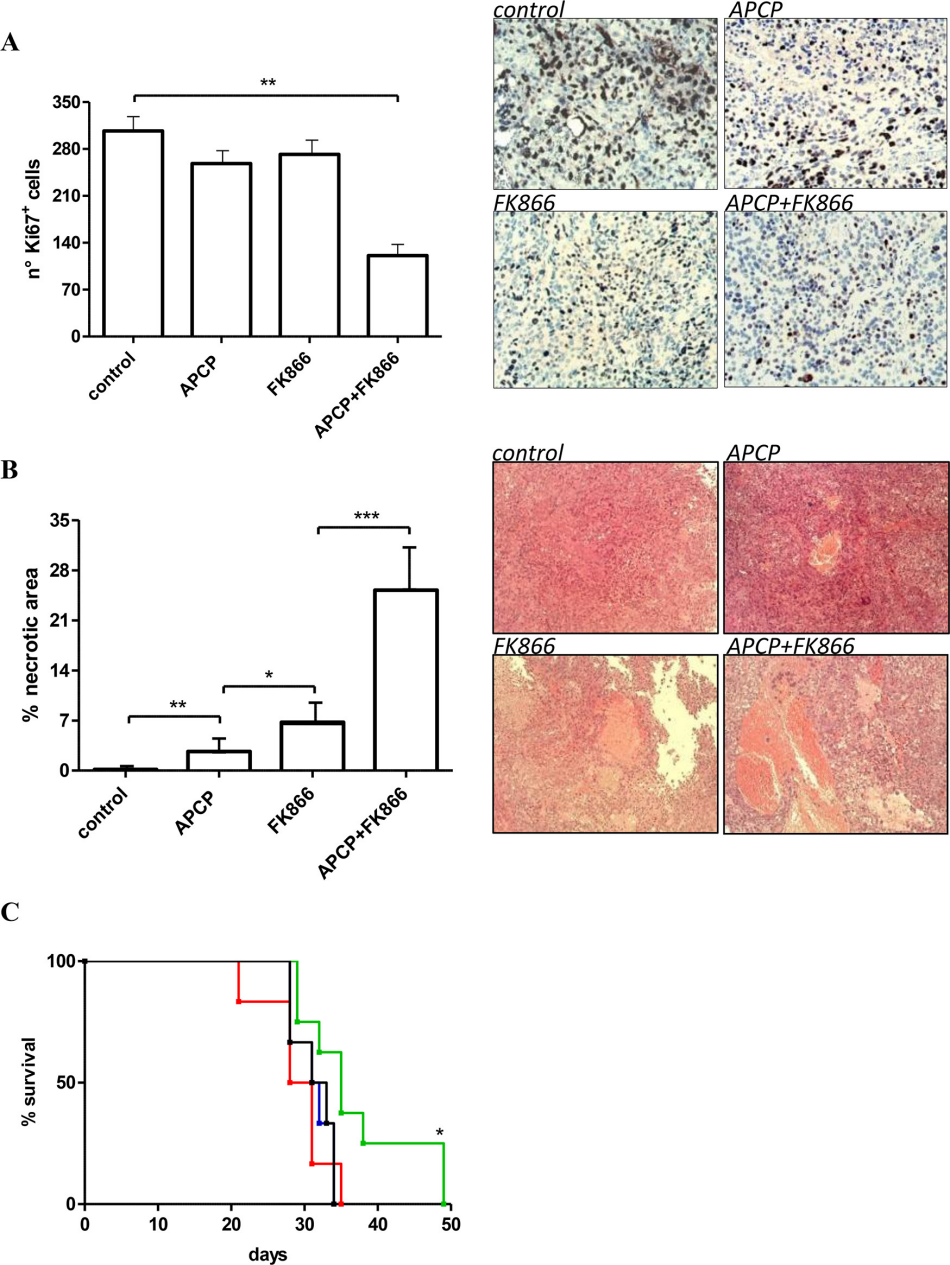
Antitumor effects of administration of FK866, APCP and FK866^+^ APCP in OVCAR- 3 bearing mice (**A**, **B**) Tumors were collected from animals treated for 28 days with vehicle (control), or FK866 or APCP or their combination: tumor tissue sections were stained with an anti-Ki67 antibody (A) or with Hematoxilyn/Eosin solution (B); the number of Ki67^+^ cells was evaluated in randomly chosen fields from different samples (*n =* 5 fields/sample); the necrotic areas were quantified using the Image J program and results represent the percentage of necrotic area relative to the total section and are the mean ± SD of at least 4 samples. *p < 0.05; **p < 0.01; ***p < 0.001. Representative images are shown. (**C**) Animals were treated with vehicle (control, black), or FK866 (blue) or APCP (red) or their combination (green) and were sacrificed when they showed signs of poor health. Survival curves were constructed by using the Kaplan–Meier method. Statistical analysis of different treatment groups was performed by Peto's log-rank test. *p < 0.05 vs black, blue and red lines.

We next aimed to evaluate the effect of combined NAMPT and CD73 inhibition on tumor cell proliferation and necrosis. To this end, we carried out immunohistochemical analyses on paraffin-embedded tissue sections of tumors from animals treated (or not) with FK866, APCP, or FK866 plus APCP for 28 days. As shown in Figure 6A, FK866 plus APCP-treated tumors contained a statistically significant lower proportion of Ki67 positive proliferating cells compared to control tumors. A representative staining with anti-Ki67 mAb of tumors from the different treatment arms is shown in Figure 6A. Histological analysis of paraffin-embedded sections from control tumor and treated tumors demonstrated that the percentage of necrosis was significantly increased in both FK866- and APCP-treated tumors, but the most potent effect was documented in the combination treatment arm (Figure 6B).

Finally, the combination FK866 plus APCP was the only treatment that significantly, albeit slightly, improved mice survival compared to vehicle (Figure 6C).

## DISCUSSION

Lowering intratumor NAD^+^ concentration is currently considered a promising strategy to treat cancer and major efforts are directed to the identification of clinically applicable NAMPT inhibitors [6–8, 13, 48]. In this study, we investigated whether the beneficial antitumor effects of NAMPT inhibition could be circumvented *in vivo* by the presence of NAD^+^ or NAD^+^ precursors, such as NMN or NR in the extracellular milieu, since these metabolites can potentially fuel NAD^+^ production through alternative pathways [25]. Several lines of evidence obtained *in vitro*, demonstrated that in OVCAR-3 cells, similarly to what previously reported for U87 and A549 cells [25], CD73 expression also generates NR from extracellular NMN, circumventing the anticancer effects of NAMPT inhibition. Specifically, the NMN-mediated rescue from FK866-induced NAD^+^ depletion and cell death were impaired by: i) CD73 silencing or inhibition with APCP (Figure 2D, 2E, 2F); ii) inhibition of NR transport (Figure 3A, 3B); iii) NRK1 silencing (Figure 3C, 3D). The fact that, in FK866-treated cells, NMN completely rescued cells from death (Figures 2E, 2F and 3B, 3D), while increasing NAD^+^ content above 10% of the control value (Figures 1C, 2D, 3A, 3C), and not to a complete restoration to values in untreated cells (i.e. approximately 20 nmol/mg proteins), is in line with the observation that cell death occurs when NAD^+^ levels are depleted to less than 5% [45].

The most relevant result obtained in this study is represented by the proof of concept that the combined inhibition of NAMPT and CD73 indeed results in a significant reduction in NAD^+^ and NMN concentration in tumors *in vivo* (Figure 4), leading to a reduced tumor cell proliferation, increased necrotic tumor cell death and to a significant, albeit small, advantage in terms of survival (Figure 6).

The results on NAD^+^ levels in tumors, heart and liver indicate that also *in vivo*, the intracellular NAD^+^ content is quantitatively related to the ectoenzymatic activity of CD73, as previously demonstrated *in vitro* in different cell lines [25]. The contribution of the different NAD^+^ biosynthetic pathways and the relevance of the utilization of extracellular NAD^+^ precursors to NAD^+^ synthesis may differ among different organs and tissues. In the heart, NAMPT inhibition determined a significant decrease in the intracellular NAD^+^ content (Figure 5), in agreement with our previous findings [46] and with our previous observation that NAMPT activity is a key contributor to NAD^+^ synthesis in this organ [49]. The concomitant inhibition of CD73 by APCP determined a further decrease of the intracellular NAD^+^ content, indicating that also in heart CD73 enzymatic activity contributes to the generation of intracellular NAD^+^. Indeed, the presence of a NRK activity has been demonstrated in this organ [49]. Conversely, in the liver, FK866 failed to cause a reduction in NAD^+^ content, in agreement with previous reports, in which the relevance of circulating levels of metabolites, other than NAM, fueling NAD^+^ synthesis in this organ is suggested, and in which the presence of a robust activity of the enzymes responsible for their conversion to NAD^+^ is proven [46, 49]. The fact that the simultaneous inhibition of CD73 and NAMPT significantly reduces (by approximately 25%) hepatic NAD^+^ content, compared to the content in FK866-treated animals (Figure 5B), indicates a contribution by CD73-mediated extracellular NR generation to NAD^+^ biosynthesis in this organ. Consistent with this notion, the presence of a NRK activity has been previously demonstrated in the liver [49] and NR supplementation to mice was found to increase NAD^+^ content in liver, muscle and brown adipose tissue [50]. Thus, NAMPT contribution to intracellular NAD^+^ levels appears to vary between different organs, tissues and tumors. In the case of the tumor cells used for this study, NAMPT and NRK seem to play a major role in NAD^+^ biosynthesis, as the simultaneous inhibition of NAMPT and CD73 significantly decreased intracellular NMN and NAD^+^ concentration (Figure 4). It is noteworthy to highlight that this result is in agreement with the data obtained by the screening that was preemptively performed to identify which NAD^+^-biosynthetic enzymatic activities are present in this cell line (Figure 1B). Indeed, this result strengthens the idea that identifying the contribution of each enzyme to NAD^+^ biosynthesis in various tissue/cell types, is crucial for the rational design of successful therapeutic strategies aimed at modulating NAD^+^ metabolism [49]. Currently, no specific inhibitors for NRK are available yet: their identification might prove useful in combined therapies to reduce NAD^+^ levels in tumors, depending on the relative contribution of this enzyme.

Our data show for the first time that NAD^+^, NMN and NR are present in ascitic exudates. NAD^+^ has been previously detected in mammalian plasma and fluids [26]. Regarding NMN, few data are available to prove its presence in murine plasma [27, 51]. This study represents the first report of NR detection in murine fluids.

The detection of the extracellular NAD^+^, NMN and NR was obviously very important for our working hypothesis. Interestingly, by inhibiting CD73 activity, an increase in extracellular NAD^+^ and/or NMN was expected (see Figure 1A): instead, this was not documented by our measurements in ascitic exudates (Figure 4). Nevertheless, the interpretation of these results is difficult since the levels of these nucleotides may also reflect the activity of ectoenzymes other than CD73, as well as the intracellular content of the same nucleotides. Indeed, NAD^+^ and NMN concentrations in the ascitic exudates seems to correlate with their intratumoral concentration (Figure 4). The marked decrease in the levels of extracellular NAD^+^ and ATP that was obtained with the combination of FK866 and APCP (Figure 4) might prove beneficial from a therapeutic prospective and also provides mechanistic insights on the well-known role of the tumor microenvironment in the control of tumor growth and tumor-related inflammation. In this context, extracellular NAD^+^ and ATP have been demonstrated to represent pro-inflammatory stimuli [52, 53]. Specifically, extracellular NAD^+^ levels in the low micromolar range, such as those detected in our ascitic exudates, effectively stimulate innate immune responses [52].

In a previous study of human ovarian cancer xenografts generated by subcutaneous injection of A2780 cells in mice, the expression of the proliferation marker, Ki67, within the tumors was significantly reduced by FK866 [54]. In our model (OVCAR-3 xenografts) the use of FK866 alone did not reduce the number of Ki-67^+^ cells, or significantly affect animal survival (possibly due to the lower dose of FK866 used in our study, i.e. 10 mg/Kg instead of 15 mg/Kg [54]). However, a reduction in tumor cell proliferation (as revealed by immunohistochemistry on tumor sections), accompanied by a consistent increase in survival, was observed in response to the co-administration of FK866 together with APCP (Figure 6A, 6C). These effects nicely correlated with the observed reductions in NAD^+^ and ATP levels. The lower number of proliferating cells in tumors from mice treated with FK866 plus APCP was also accompanied by the presence of larger necrotic areas (Figure 6B).

CD73 is emerging as an appealing target in cancer therapy, especially for its key role as an immunosuppressive factor within the tumor microenvironment. Small molecular inhibitors and monoclonal antibodies against CD73 have been recently developed [55, 56], and our data indicate that combinations of NAMPT inhibitors and CD73-targeting agents might prove clinically effective.

Regarding the translational relevance of our results, recently it has been demonstrated that, in line with what reported for other tumor tissues [30–37, 57], CD73 is associated with poor prognosis in high-grade serous ovarian cancer, representing a prognostic marker of patient survival [58]. From a study on patients affected by EOC (subdivided on the basis of different clinicopathologic variables), it was inferred that tumors exhibit various levels of CD73 expression. In that study, 29.9% of the patients had CD73^−^ tumors, and the rest had a variable level of CD73 expression, with CD73 being more frequently expressed in mucinous and in clear cell carcinoma, compared to serous and endometriod adenocarcinoma [59]. Thus, a personalized treatment approach that takes into account CD73 expression could be considered. Unexpectedly, in the latter study, the CD73^+^ ovarian cancer group (mostly including patients with mucinous adenocarcinoma) showed better prognosis and the authors speculated that cell differentiation stage had more influence on the prognosis than the adverse effect of CD73 [59].

In another clinical study, it was demonstrated that CD73 is highly expressed by mesenchymal-like and also, to a lower level, by epithelial-like cells isolated from ascites or cancer tissue from patients with EOC [60]. In fact, an advantage of the combination of NAMPT inhibitors with CD73 inhibitors is that their synergism could be achieved regardless of whether NR production from extracellular NAD^+^/NMN is catalyzed by CD73 harbored on epithelial or on mesenchymal-like cells. Finally, it was reported that CD117^+^/CD73^+^ fibroblast-like stromal cells are significantly associated with poor clinical manifestations and poor survival probability in ovarian carcinomas, and further studies of CD73^+^ stromal cell-targeting therapies in EOCs were thought to be important [38]. Thus, targeting CD73 could prove beneficial under several respects, directly targeting NAD^+^ biosynthesis in the malignant epithelial cells (as those used in our study), but also preventing CD73 activity on the stromal cells that are present within the tumor, which might contribute to the generation of NR and adenosine in the tumor extracellular microenvironment and thereby promote tumor growth [61].

In conclusion, our study demonstrates that the CD73 inhibitor APCP can enhance the antitumor activity of FK866 in ovarian cancer cells. The potentiation of FK866 with APCP occurs through an enhancement of the depletion of NAD^+^ and ATP that is initiated by FK866, ultimately resulting in tumor growth inhibition and necrotic tumor cell death. This study provides the proof-of-concept that combining NAMPT and CD73 inhibitors might prove clinically beneficial and warrants further investigations of this approach in other models and then, potentially, in clinical trials.

## MATERIALS AND METHODS

### Drugs

All chemicals were purchased from Sigma (Milan, Italy), unless otherwise stated. FK866 was in part generously provided by the NIMH Chemical Synthesis and Drug Supply Program and in part purchased from Sigma. NR was enzymatically synthesized from NAD^+^ as described [25].

### Cell culture

OVCAR-3 cells (human ovarian cancer cells), transfected with fire fly-luciferase, obtained as described in [62] were grown in RPMI1640 supplemented with 10% fetal calf serum, penicillin (100 U/ml) and streptomycin (100 μg/ml) at 37°C in a humidified atmosphere with 5% CO_2_.

### CD73 and NRK1 siRNA gene silencing

OVCAR-3 cells were transfected with Stealth^™^ RNAi (Life Technologies Italia, Monza, Italy) targeting human CD73 (Oligo ID: HSS101578) or with Mission esiRNA (Sigma) targeting human NRK1 (Oligo ID: HU-09786–1). Cells were electroporated with 2 μM duplex siRNA with the Nucleofector System, using the Cell Line Nucleofector Kit V, program V-005, following the manufacturer's instructions. As control, OVCAR-3 cells were transfected with Stealth^™^ RNAi Negative Control Duplex (Life Technologies Italia) for CD73 or with Mission negative control (Sigma) for NRK1. Cells were seeded as follows: 2×10^5^ onto 35×10 mm tissue culture dishes for mRNA quantification; 2×10^5^ cells/well in 12-well plates for the determination of intracellular NAD^+^ levels; 5×10^3^ cells/well in 96-well plates for viability assays. All experiments were carried out 24 h after transfection.

### qPCR analyses

Twenty-four h after transfection, total RNA was extracted from OVCAR-3 cells using the RNeasy Micro Plus Kit (Qiagen, Milan, Italy). Quality and quantity of RNA were analyzed using a NanoDrop spectrophotometer (Nanodrop Technologies, Wilmington, DE). cDNA (0.5 μg) was synthesized by using iScript cDNA Synthesis Kit (Bio-Rad Laboratories, Milan, Italy). PCR primers were designed by using through Beacon Designer 2.0 Software (Bio-Rad) and their sequences were as follows: hCD73: 5′-GCTCGGCTCTTCACCAAG-3′ (forward), 5′-TCAGT CCTTCCACACCATTAT-3′ (reverse); hNRK1: 5′-AAG CCCCTTGACACTATATGGA-3′ (forward), 5′-TCTGG AGGCTGATAGACCCT-3′ (reverse); hGAPDH: 5′-CCTG TTCGACAGTCAGCCG-3′ (forward), 5′-CGACCAAA TCCGTTGACTCC-3′ (reverse); hHPRT1: 5′-GGTCA GGCAGTATAATCCAAAG-3′ (forward), 5′-TTCATTAT AGTCAAGGGCATATCC-3′ (reverse).

Statistical analyses of the qPCR were obtained using iQ5 Optical System Softwar version 2.0 (Bio-Rad) based on the 2^−ΔΔ^Ct method, which calculated relative changes in gene expression of the target normalized to GAPDH and HPRT-1. Experiments were repeated 3 times in triplicate.

### Western blot analyses

Twenty-four h after transfection, level of CD73 expression were evaluated on cell lysates from control and silenced cells by Western Blot analysis, performed as described in [25] and visualized with the following antibodies: anti-CD73 (SAB1306278–40TST, Sigma), anti-vinculin (kindly provided by Prof. E. Turco, University of Torino, Italy). Western blots were developed with the ECL-PLUS kit (GE Healthcare), according to the manufacturer's instructions. Band detection and densitometry were performed using the Chemi-Doc System and the quantity one software package (Bio-Rad).

### Determination of intracellular NAD^+^ levels

OVCAR-3 cells (not transfected, transfected with negative control siRNA or with siRNA targeting CD73 or NRK1) were plated at a density of 10^5^ cells/well in 12-well plates and cultured in 500 μl of complete RPMI1640 in the presence or absence of 30 nM FK866 and, depending on the experimental setting, with or without 5 μM dypiridamole, and supplemented twice a day (at 9 am and at 6 pm) for 2 days, with or without 10 μM of NMN or NR. Then, cells were harvested and lysed in 0.05 ml of 0.6 M PCA at 4°C. Cell extracts were centrifuged for 3 min at 16,000 x g, the supernatants were collected and an aliquot was diluted 20-fold in 100 mM sodium phosphate buffer, pH 8.0, for determination of NAD^+^ content, as described [63]. NAD^+^ values were normalized to protein concentrations, determined by Bradford assay [64].

### Cell viability assays

OVCAR-3 cells (5×10^3^/well in 96-well plates; not transfected, transfected with negative control siRNA or with siRNA targeting CD73 or NRK1) were incubated in the presence or absence of 30 nM FK866, with or without 1 μM adenosine 5′-(α, β-methylene)diphosphate (APCP), with or without 5 μM dypiridamole, and, supplemented (or not) twice a day (at 9 am and at 6 pm) for 2–3 days, with or without 10 μM NMN or NR. Cell viability was measured as described [25].

### Assays of ectocellular AMP-degrading enzyme activity

The ectocellular enzymatic activity of CD73 was assayed by incubating not transfected, transfected with negative control siRNA or with CD73-targeting siRNA, OVCAR-3 intact cells (0.5×10^6^ in 0.5 ml of phosphate buffered saline, PBS) in the presence of the substrate AMP (0.2 mM). At various times (0, 1, 2.5 and 5 min), 100-μl aliquots of the incubations were withdrawn, briefly centrifuged, and 2.5% trichloroacetic acid (TCA) was added to 90-μl supernatants. Samples were centrifuged and the excess TCA removed with diethylether. The amounts of adenosine produced were determined by the phosphate HPLC analysis described previously [25].

### Assay of ectocellular CD38 activity on NAD^+^ or NGD^+^

Ectocellular enzymatic activities of CD38 were assayed by incubating 0.5×10^6^ OVCAR-3 intact cells in 0.5 ml of PBS in the presence of the substrates NAD^+^ or NGD^+^ (0.5 mM). At various times (0, 10 and 60 min), 100-μl aliquots of the incubations were withdrawn, briefly centrifuged, and 2.5% trichloroacetic acid (TCA) was added to 90-μl supernatants. Samples were centrifuged and the excess TCA removed with diethylether. The production of ADPR (from NAD^+^) or cGDPR (from NGD^+^) were determined by the phosphate HPLC analysis described previously [63].

### Assays of NAMPT, NRK, NAPRT and QAPRT activities

OVCAR-3 cells were incubated (or not) with 30 nM FK866 for 24 h. Approximately 5×10^6^ OVCAR-3 cell pellets were homogenized with 0.2 ml buffer consisting of 50 mM TRIS/HCl, pH 7.5, 0.15 M NaCl, 1 mM dithiothreitol, 1 mM phenylmethanesulfonyl fluoride, and 0.002 mg/ml each of leupeptin, antipain, chymostatin and pepstatin. Homogenates were centrifuged at 20,000 x g for 10 min at 4°C, and the supernatants were immediately used for the assays of enzymatic activities. The simultaneous measurement of the four enzymatic activities was performed by using the coupled fluorometric assay described in [49]. The enzymes' activity was referred to the protein concentration determined according to [63].

### Flow cytometric analysis

OVCAR-3 cells (0.4 × 106) were harvested, washed once with PBS buffer and incubated for 20 min at 37°C in the presence (or absence) of the anti-CD73 antibody (Sc130006, Santa Cruz Biotechnology, Inc., Santa Cruz, CA, USA), in PBS containing 0.5% FBS. Cells were washed once with PBS, and incubated for 20 min at 37°C in the presence of an anti-mouse secondary antibody conjugated with Alexa-576, in PBS containing 0.5% FBS. Cells were washed twice with PBS and analyzed using a FACS Calibur flow cytometer (Becton Dickinson) by acquiring 10,000 events.

### *In vivo* treatment

Athymic nude mice were intraperitoneally (ip) inoculated with 1.5×10^5^ OVCAR-3 cells transfected with firefly luciferase. The *in vivo* bioluminescence using IVIS™ Imaging System, performed 5 days after inoculation, allowed us to monitor tumor development and randomize the mice in the different groups of treatment. Thus, animals were divided in four groups, treated as follows: vehicle (0.05% dimethyl sulfoxide, DMSO), FK866 (10 mg/Kg administered ip once a day for 28 days), APCP (15 mg/Kg administered ip every other day for 28 days) or the combination of the two molecules. On the day of sacrifice, tumor samples, heart and liver were collected and fixed in 20% buffered formalin, routinely processed and embedded in paraffin. One part of some of the lesions was embedded in OCT, frozen in liquid nitrogen and stored at −80°C. Ascites exudates were collected, centrifuged to discard possible cells and immediately frozen in liquid nitrogen. In a second trial, conducted as above, animals were sacrificed when they showed signs of poor health to evaluate survival.

### Determination of NAD^+^, NMN, ATP and NR levels in samples from treated animals

Nucleotides were extracted from tumor, liver and heart as described in [46]. Perchloric acid (PCA, 0.6 M, final concentration, for NAD^+^, NMN and NR determination) or trichloroacetic acid (TCA, 5% v/v, final concentration, for ATP determination) was added to the ascitic exudates for deproteinization: samples were then neutralized by adding K_2_CO_3_ (for PCA) or by extraction with diethyl ether (for TCA). NAD^+^ and ATP levels in tumors were determined as described [46], and normalized to tissue weight (for tumor, liver and heart). ATP levels in ascitic exudates were measured on neutralized extracts with an ATP determination kit (Invitrogen, Carlsbad, CA) following the manufacturer's instruction. Twenty-μl of the extract were added to 180 μl of reaction buffer in each well of a 96-well plate. Luminescence was measured using a FLUOstar OPTIMA (BMG Labtech, Ortenberg, Germany) and ATP concentrations in the samples were calculated from the ATP standard curve.

NMN and NR were measured upon derivatization with acetophenone and spectrofluorometric HPLC analysis. Derivatization was performed by mixing 50 μl of the nucleotide extracts with 100 μl KOH 1N and 50 μl acetophenone. After 15 min incubation at 4°C, 100 μl of formic acid was added and the samples were heated for 5 min at 100°C. The spectrofluorometric HPLC analysis was carried out as described in [65]. Measurements were performed by using a duplicate sample analyzed in parallel and containing known amounts of NMN and NR spikes.

### Immunohistochemical staining of tissues

Immunohistochemical staining of tissue sections was performed using the Envision System HRP mouse (Dako). Briefly, 5 mm thick sections were cut from formalin fixed, paraffin embedded blocks, deparaffinized with xylene and rehydrated by passages through decreasing concentrations of ethanol (from 100% to 70%). Endogenous peroxidase activity was blocked by 30 min incubation at room temperature with methanol containing 3% H_2_O_2_. Tissue sections were then incubated at 98°C for 40 min in EDTA 1 mM (pH 8.0) for antigen retrieval (Sigma). After rinsing in PBS (Sigma), tissue sections were incubated 1 hour at Room Temperature with anti-Ki67 (Dako). Tissue sections were washed twice in PBS and incubated for 30 min at room temperature with Dako Envision System horse radish peroxidase (HRP) Mouse. After washing in PBS, peroxidase activity was detected by incubating tissue sections for 6–10 min at room temperature with Dako Liquid DAB Substrate Chromogen System (Dako). Tissue sections were counterstained with Mayer's hematoxylin (Sigma). The number of positively stained tumor cells in each lesion was evaluated independently by two investigators. The variation between the results obtained by the two investigators was less than 10%.

Some sections were processed and stained with Hematoxylin/Eosin solution and the necrotic areas were quantified using the Image J program.

### Statistical analyses

All parameters were tested by paired *t* test or by Two-tailed unpaired *t* test with 95% Confidence Interval; *p* values < 0.05 were considered significant. In the Figures, only relevant comparisons are shown. The statistical significance of differential survival between experimental groups of animals was determined by Kaplan-Meier curves and Peto's log-rank test by the use of StatDirect statistical software (CamCode, Ashwell, UK). All statistical tests were two-sided, and *P* values less than .05 were considered statistically significant.

## SUPPLEMENTARY MATERIALS FIGURES


