# Supplementary material for: Antitumor effect of combined NAMPT and CD73 inhibition in an ovarian cancer model
Source: Oncotarget. 2015 Dec 8;7(3):2968–84. doi: 10.18632/oncotarget.6502 (PMC4823084; doi:10.18632/oncotarget.6502)
Supplement: Supplementary file 1 [file oncotarget-07-2968-s001.pdf]

Antitumor effect of combined NAMPT and CD73 inhibition in an ovarian cancer model

Supplementary Materials

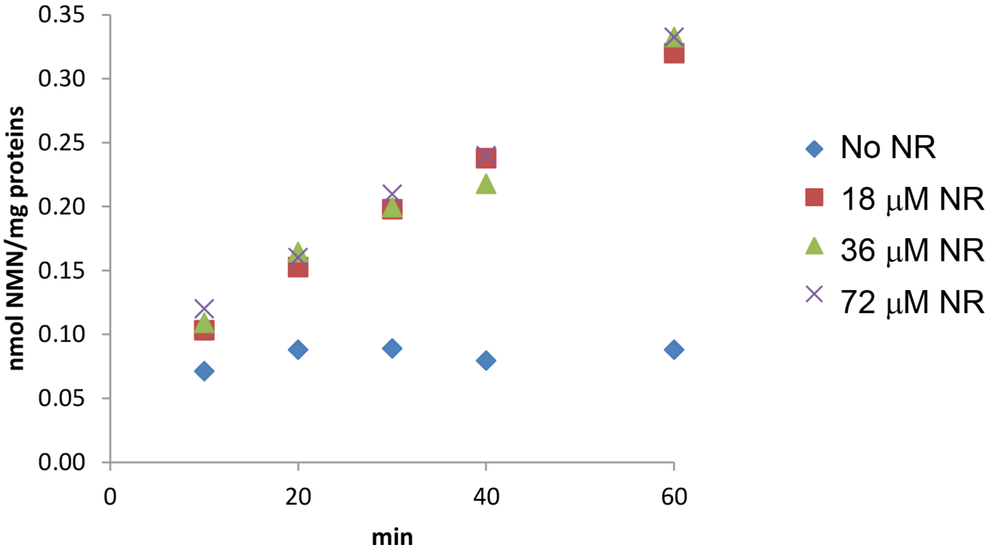

Supplementary Figure S1: NRK enzymatic activity in the presence of different NR concentration. NRK activity was evaluated on OVCAR-3 cell lysate in the prensence of 0, 18, 36, 72 µM NR, as indicated: the production of NMN was measured. One representative assay is shown. The NRK activity was approximately 0.24 nmol NMN/h/mg proteins at all the used substrate concentrations.

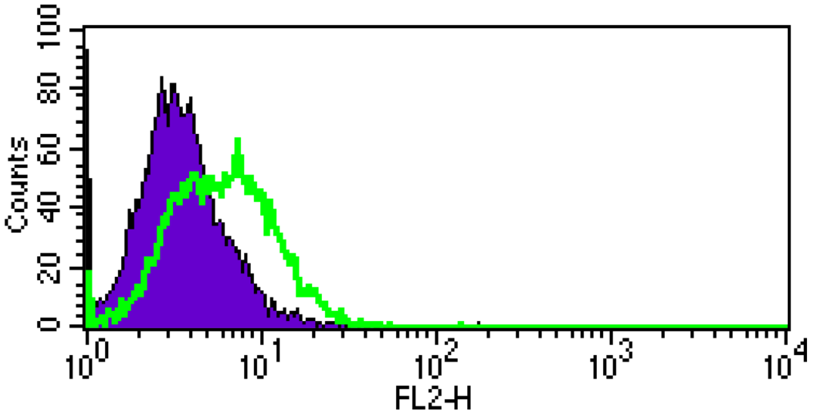

Supplementary Figure S2: FACS analysis. OVCAR-3 cells incubated in the presence (green line), or absence (solid purple) of the anti-CD73 antibody (Sc130006, Santa Cruz Biotechnology), in PBS containing 0.5% FBS. Cells were washed once and incubated in the presence of an anti-mouse secondary antibody conjugated with Alexa-576, in PBS containing 0.5% FBS. Cellular fluorescence was measured in 10,000 cells. A representative analysis is shown.
